# Supplementary figures and images for: The Novel SSTR3 Agonist ITF2984 Exerts Antimitotic and Proapoptotic Effects in Human Non-Functioning Pituitary Neuroendocrine Tumor (NF-PitNET) Cells
Source: Int J Mol Sci. 2024 Mar 23;25(7):3606. doi: 10.3390/ijms25073606 (PMC11011875; doi:10.3390/ijms25073606)

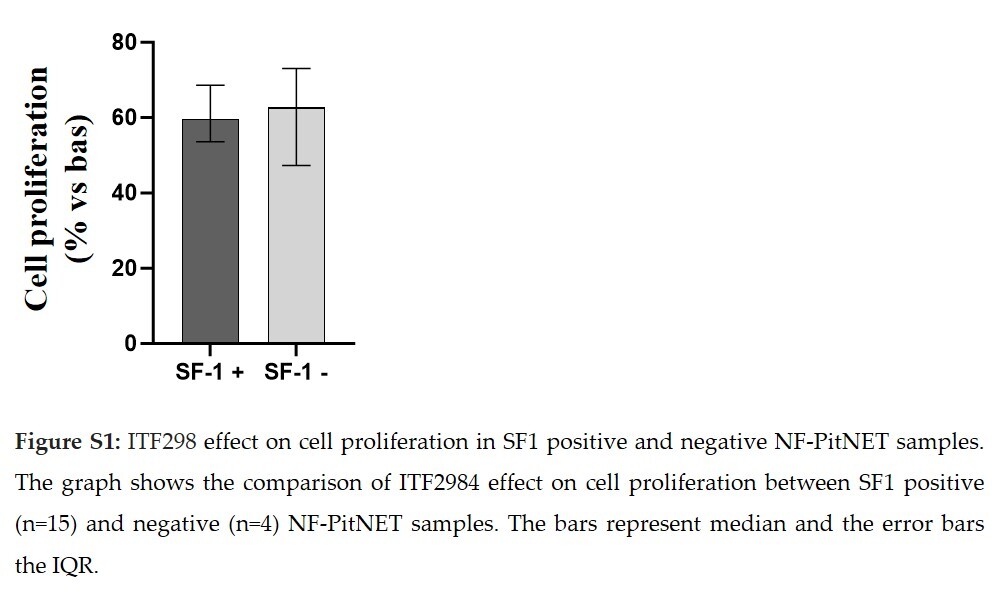

Supplement: Supplementary file 1 [file ijms-25-03606-s001.zip › Supplementary Figure S1.jpg]

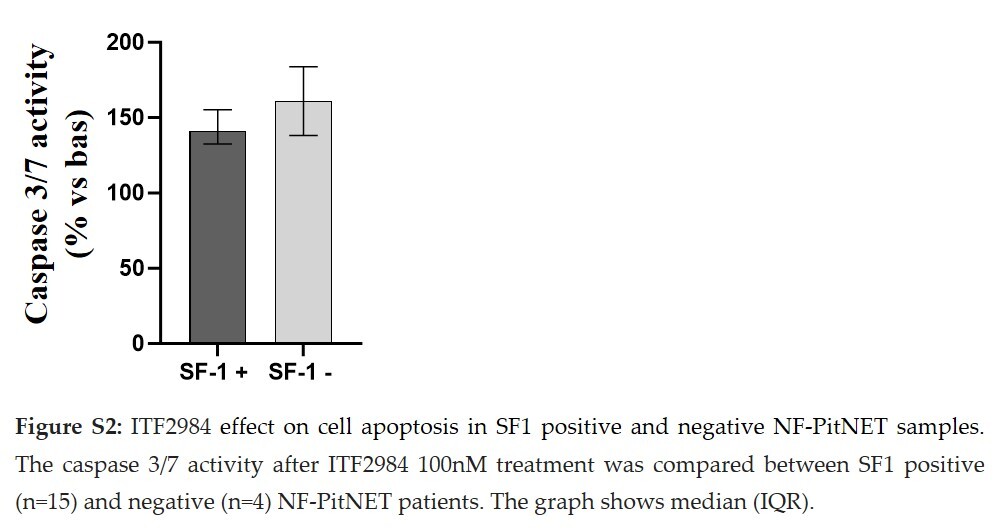

Supplement: Supplementary file 1 [file ijms-25-03606-s001.zip › Supplementary Figure S2.jpg]

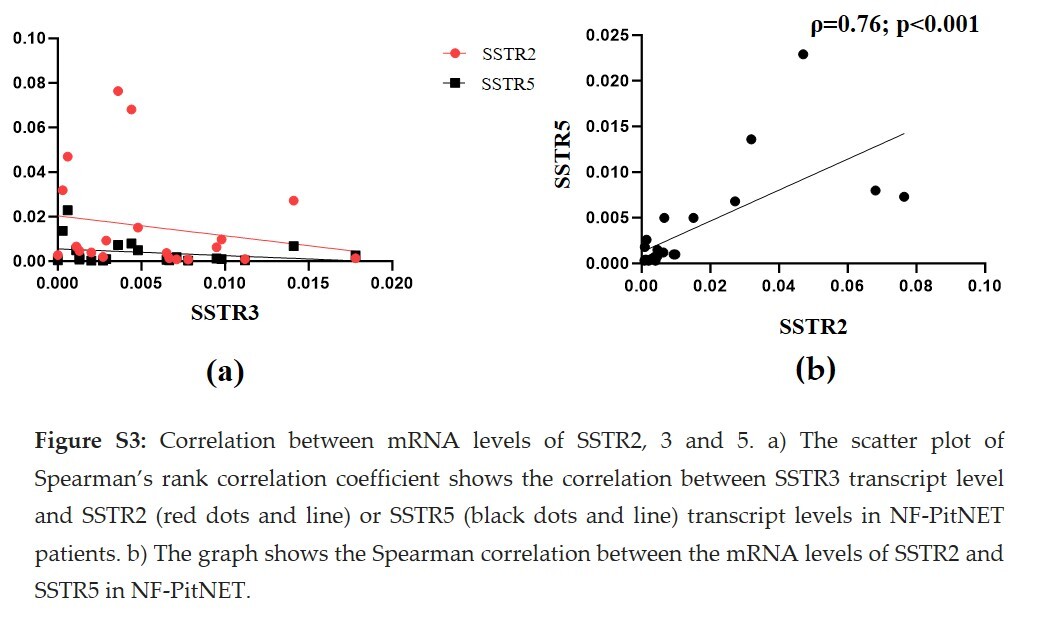

Supplement: Supplementary file 1 [file ijms-25-03606-s001.zip › Supplementary Figure S3.jpg]

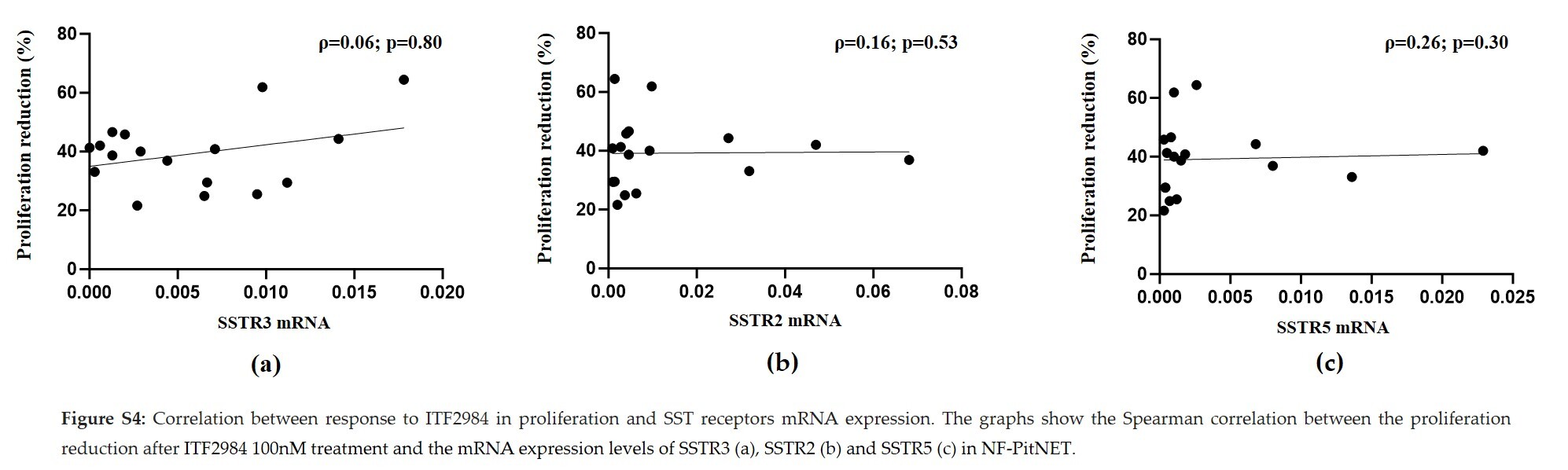

Supplement: Supplementary file 1 [file ijms-25-03606-s001.zip › Supplementary Figure S4.jpg]

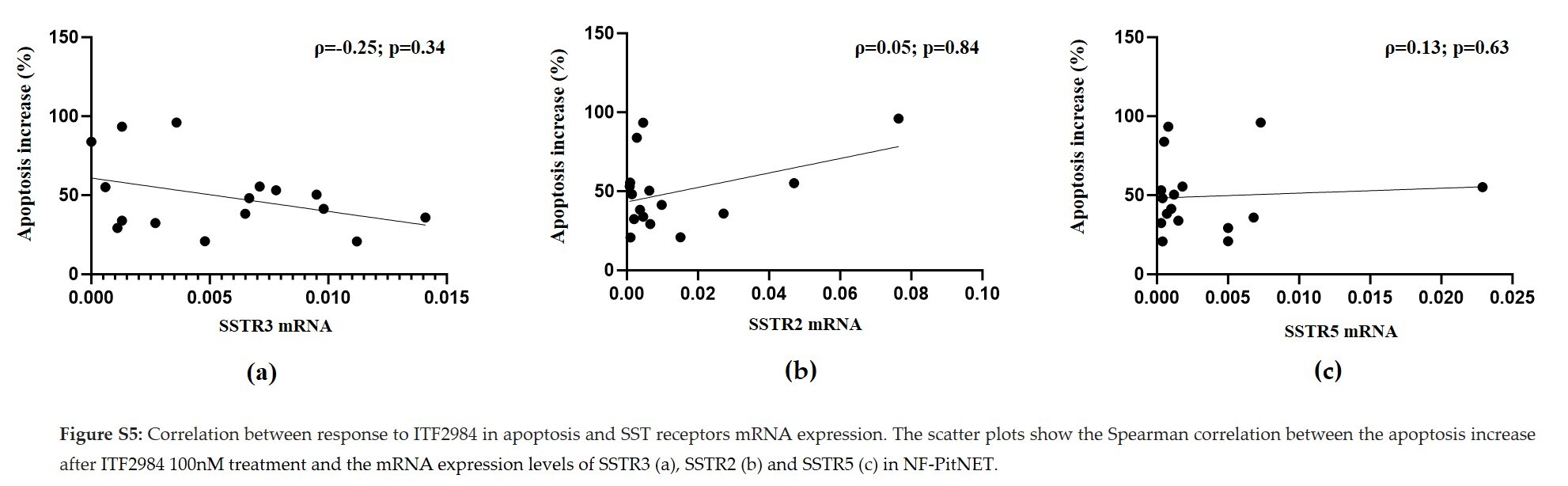

Supplement: Supplementary file 1 [file ijms-25-03606-s001.zip › Supplementary Figure S5.jpg]

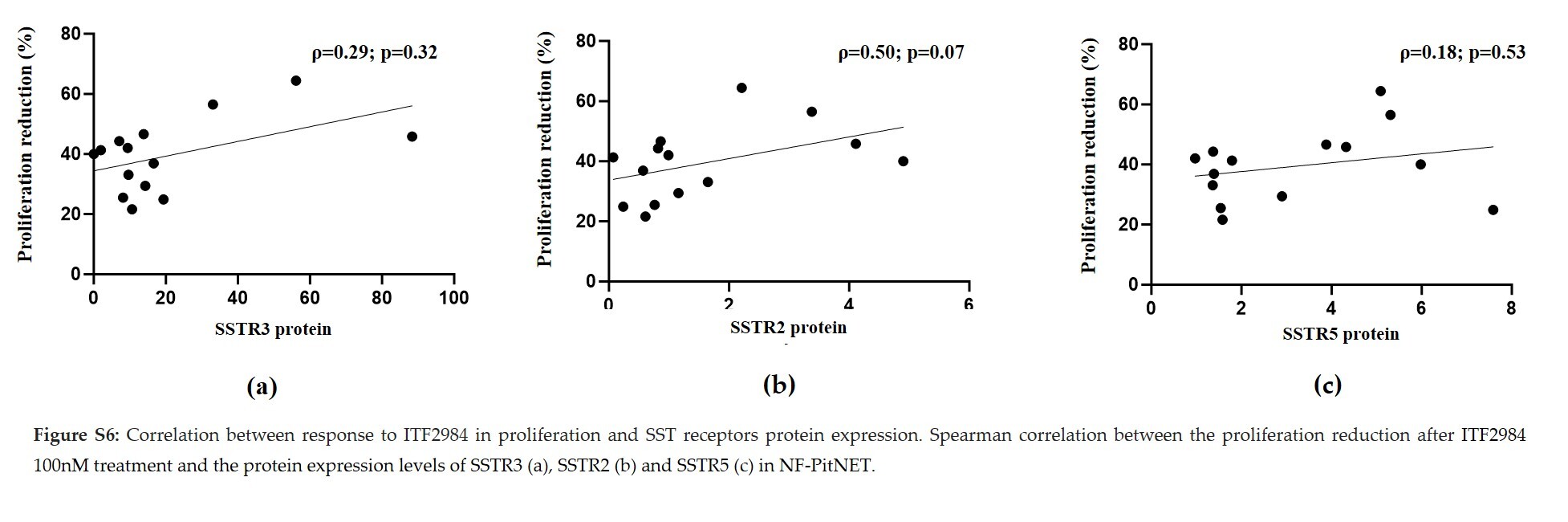

Supplement: Supplementary file 1 [file ijms-25-03606-s001.zip › Supplementary Figure S6.jpg]

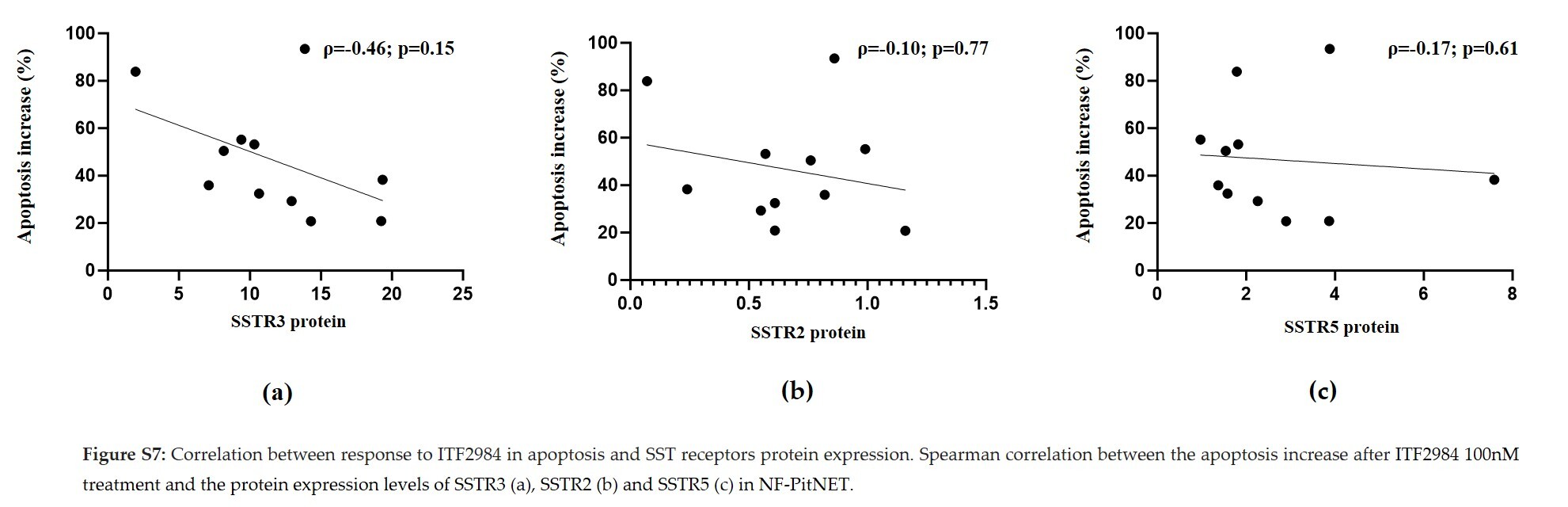

Supplement: Supplementary file 1 [file ijms-25-03606-s001.zip › Supplementary Figure S7.jpg]

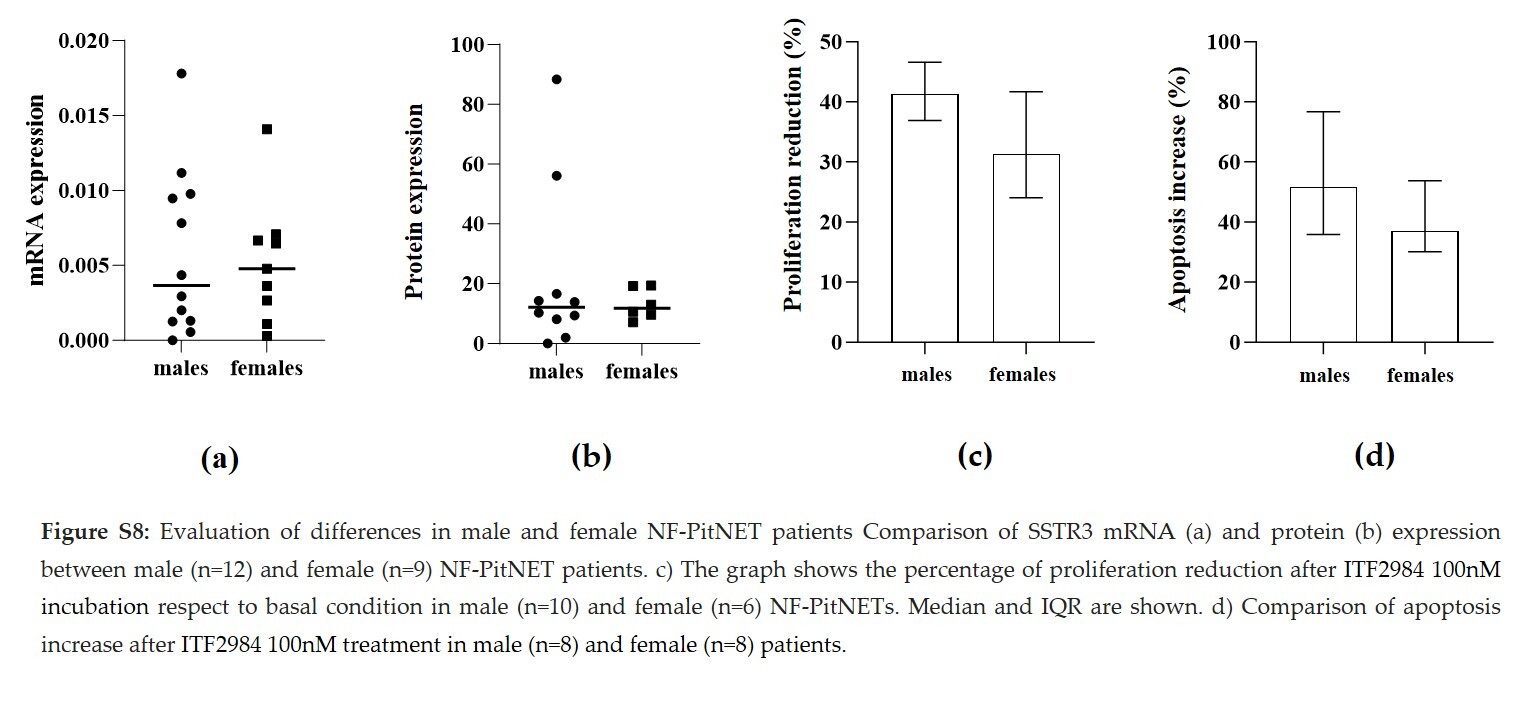

Supplement: Supplementary file 1 [file ijms-25-03606-s001.zip › Supplementary Figure S8.jpg]

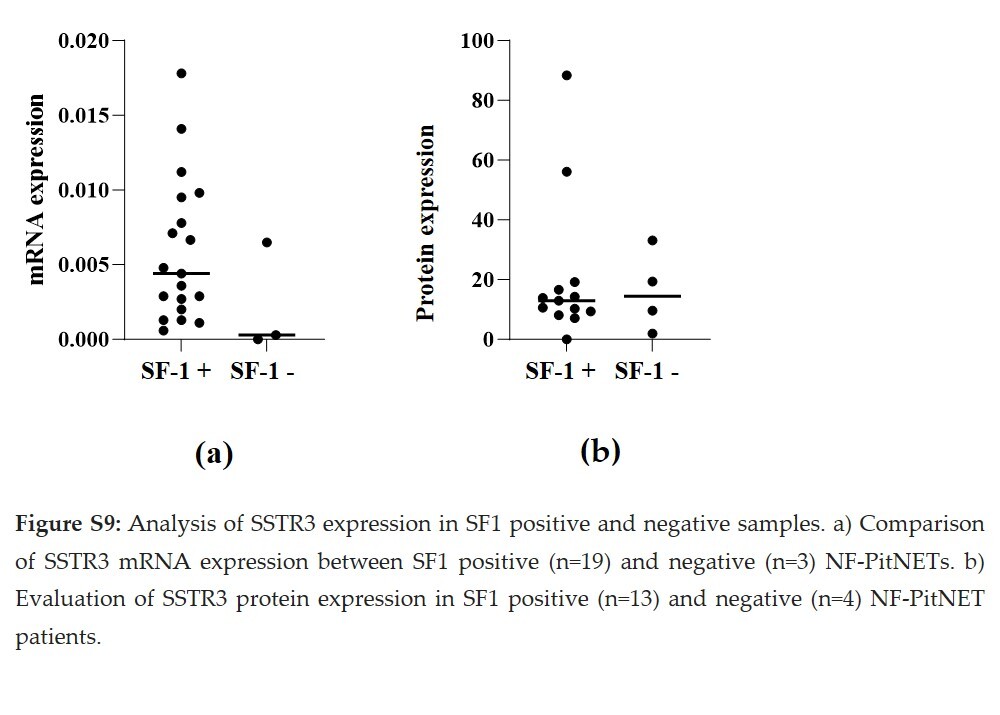

Supplement: Supplementary file 1 [file ijms-25-03606-s001.zip › Supplementary Figure S9.jpg]
